# Dietary assessment methodologies in eating disorders: a pilot comparative validation study exploring the diet history method

**DOI:** 10.1186/s40337-025-01392-x

**Published:** 2025-09-29

**Authors:** Melissa Hart, Kirrilly Pursey, Tracy Burrows, David Sibbritt

**Affiliations:** 1 College of Health, Medicine and Wellbeing, University of Newcastle, Callaghan, NSW 2308 Australia; 2 Hunter New England Mental Health Service, Waratah, NSW 2298 Australia; 3 Hunter Medical Research Institute, New Lambton Heights, NSW 2305 Australia; 4 Western Sydney University, Penrith, NSW 2751 Australia; 5 University of Technology Sydney, Ultimo, NSW 2007 Australia

**Keywords:** Dietary assessment, Diet history, Dietary recall, Dietary validation, Eating disorders

## Abstract

**Objective:**

Dietary intake and sub-optimal nutrition have a significant impact on clinical risk, outcomes and mortality in people with an eating disorder. Despite the range of dietary patterns and nutritional issues, there is limited evidence to guide effective use of dietary assessment methods. The aim of this pilot study was to examine the validity of the diet history against routine nutritional biomarkers in female adults with an eating disorder attending a regional outpatient service.

**Method:**

This secondary data analysis utilised demographics, nutrient intakes from diet history and nutritional biomarker data. Spearman’s rank correlation, simple and quadratic weighted kappa statistics and the Bland-Altman method were used to explore validity of the diet history compared to nutritional biomarkers.

**Results:**

Thirteen female participants (median age 24 years; median BMI 19 kg/m^2^) with an eating disorder were included. Energy-adjusted dietary cholesterol and serum triglycerides showed moderate agreement (simple kappa K = 0.56, *p* = 0.04), and dietary iron and serum total iron-binding capacity showed moderate-good agreement (simple kappa K = 0.48, *p* = 0.04; weighted kappa K = 0.68, *p* = 0.03). Dietary iron and serum total iron-binding capacity were only significantly correlated when dietary supplements were included (*r* = 0.89, *p* = 0.02). Dietary estimates of protein and iron improved with larger intakes.

**Conclusions:**

This pilot study suggests the diet history may be useful for assessing dietary intake in people with an eating disorder and highlights the importance of targeted questioning around dietary supplement use. Determining the validity of dietary assessment methods across ages, diagnoses and settings is required.

**Supplementary Information:**

The online version contains supplementary material available at 10.1186/s40337-025-01392-x.

## Background

Eating disorders are mental illnesses associated with significant morbidity and mortality [1]. Compromised nutritional status is a key feature and can contribute significantly to morbidity and clinical risk [2]. To minimise nutrition-related complications and optimise treatment outcomes, accurate assessment of dietary intake and nutritional status is an important care component. Very few studies have, however, investigated the validity of dietary assessment methods in eating disorders, including the diet history, despite the known alterations in eating and exercise behavior, such as dietary restriction, binge eating, purging, excessive physical activity and the frequent use of dietary supplements and substances to influence weight or shape.

Administration of the diet history is a common clinical practice in the assessment of nutritional status and eating behaviour of people with an eating disorder. Information gathered is used to direct nutrition intervention, including around nutritional rehabilitation, particular foods consumed, behaviours around eating or food beliefs. The Burke diet history [3] assesses individual food consumption and nutrient intakes, and can produce a more complete and detailed description of food intake than food records, a single 24-hour recall or food frequency questionnaires [4, 5]. Habitual intake from core food groups, specific dietary items, attitudes and beliefs, and behaviours such as missed meals, dieting and non-dieting days (particularly relevant to eating disorders) are assessed. Important disadvantages of diet histories are recall bias and social desirability bias [6]. Energy and most nutrients can be estimated reasonably accurately [6], though bias and error due to reliance on participant memory, conceptualization of portion sizes, interviewer bias and tendencies to over-estimate energy intakes are known [4, 5].

Cognitive function impacts the ability to accurately describe food portion sizes and frequency of consumption, and starvation symptoms are known to impact cognitive function in eating disorders [7]. Binge eating episodes are often highly stressful situations involving loss of control and consumption of large amounts of food in a short period of time, which may influence episodic memory of the type and quantity of food consumed during binge episodes. Other features (including dietary restriction, ritualistic eating behaviours, discomfort around disclosing behaviours, compensatory behaviours and use of supplements and other substances) may contribute to bias and error. Data from diet histories rely heavily on the skill of the interviewer, particularly in reducing over-reporting or under-reporting, and observer bias can occur. Dietitians are trained in administration of diet histories, which can reduce error compared to non-trained clinicians.

The accuracy of the diet history and other dietary assessment methods in assessing dietary intake and nutritional status in people with an eating disorder remains unclear. One study using the diet history method in anorexia nervosa (AN) found macronutrient and micronutrient intakes correlated with observed intake [8]. Studies using other dietary assessment methods compared to direct observation have reported mixed findings. Studies of macronutrient intake using the 24-hour recall method compared to observation found in adults with bulimia nervosa (BN) over-estimation as dietary intake increases [9], in women with binge eating disorder (BED) either underreporting [10] or moderate agreement [11], and in a sample of people with AN or BN high correlation [12]. A study using the food record method found over-reporting of intake in AN, particularly at increasing levels of intake [13]. Overall, these studies involved small sample sizes (*n* = 15–30), were conducted in people of differing ages and diagnoses, and covered differing time periods for assessing dietary intake, limiting comparability of the diet history study with other methods. The use of laboratory or inpatient observations as comparison methods raises limitations and limits generalizability to naturalistic settings. Blood tests can be used to overcome these limitations and are used to examine the validity of subjective methods of dietary assessment. Specific biomarkers can provide objective measures of dietary exposure and nutritional status if sensitive and a time integrated reflection of nutrient intake, although selection must be made on a nutrient-by-nutrient basis [14].

Despite the widespread use of the diet history to inform eating disorders treatment recommendations and nutritional monitoring, evidence for understanding the level and impact of measurement error is lacking. Opportunities to explore diet history validation studies in naturalistic settings and utilizing cost-effective strategies such as use of routine data are also required to inform clinical practice. The aim of this study was to undertake a pilot comparative validity study to examine the relationship between nutritional intake assessed by diet history and nutritional biomarkers collected in routine clinical practice in female adults with an eating disorder attending a regional outpatient service.

## Methods

This pilot study was conducted using secondary data collected at a public eating disorders outpatient service in a regional area in New South Wales, Australia. Data were collected by the clinical dietitian and included patient demographics, diagnosis, self-reported disordered eating behaviours, anthropometry, diet history data and nutritional biomarkers from blood tests. Participants completed signed, informed consent to participate. Ethics approval was obtained from the Hunter Area Research Ethics Committee (Number 02/12/11/3.22). Patients were eligible for inclusion if they were female, aged 18–64 years, had an eating disorder diagnosis according to the Diagnostic and Statistical Manual of Mental Disorders, Fourth Edition [15], and attended the outpatient clinic in person for assessment within the study period. Additional File 1 provides further details regarding methodology.

Nutritional biomarkers selected from the available routine blood tests included cholesterol, triglycerides, protein, albumin, iron, haemoglobin, ferritin, total iron-binding capacity (TIBC) and red cell folate, and were collected via blood test within 7 days prior to diet history administration. Daily nutrient intake data selected for comparability to available nutritional biomarkers included saturated fat, cholesterol, protein, iron and folate. Statistical analysis was conducted using STATA v8.2 [16]. Nutrients were adjusted for energy. Spearman’s rank correlation coefficients, simple and quadratic weighted kappa statistics and Bland-Altman analyses were conducted. Kappa statistics were interpreted as poor (≤ 0.2), fair (> 0.2 and ≤ 0.4), moderate (> 0.4 and ≤ 0.6), good (> 0.6 and ≤ 0.8) or very good (> 0.8 and ≤ 1) [17]. Additional File 1 provides further details regarding analyses.

## Results

Thirteen participants with a median age of 24 years (standard deviation 6) and median BMI of 19 kg/m^2^ (standard deviation 5.8) were included in this study (Table 1 and Additional File 1 Fig. 1). Four participants had AN, one BN and eight Eating Disorders Not Otherwise Specified (EDNOS). Five participants reported binge eating (38%), six self-induced vomiting (46%) and five laxative use (38%). Physical activity level (PAL) was reported as light in most participants (69%). Seven (54%) reported using a nutritional supplement. Additional File 1 shows additional descriptive data.

**Table 1:** Participant characteristics and daily nutrient intakes (*n* = 13)

	AN and BN (*n* = 5)	EDNOS (*n* = 8)	All Subjects (*n* = 13)
***Characteristic***			
Age in years median (IQR)	24 (12)	24 (7)	24 (7)
BMI kg/m^2^ median (IQR)	17 (1)	19 (7)	19 (3)
Binge Eating *n* (%)	4 (80)	1 (13)	5 (38)
Vomiting *n* (%)	4 (80)	2 (25)	6 (46)
Laxative use *n* (%)	2 (40)	3 (38)	5 (38)
BMR kJ/day median (IQR)	5111 (248)	5353 (1488)	5217 (1110)
EEE kJ/day median (IQR)	8489 (1243)	8695 (2754)	8489 (2771)
***Daily intake of macronutrients and selected micronutrients*** - ***median (IQR)***			
Energy (EEI) (kJ)	9404 (10063)	4763 (5547)	4160 (5720)
Total fat (g)	27 (34)	20 (63)	21 (63)
Saturated fat (g)	12 (10)	7 (22)	7 (22)
Cholesterol (mg)	96 (42)	57 (180)	61 (183)
Protein (g)	43 (55)	57 (51)	54 (51)
Carbohydrate (g)	374 (619)	173 (250)	150 (225)
Iron (mg)	9 (17)	6 (5)	7 (7)
Folate (ug)	280 (54)	95 (228)	245 (285)

Supplement use included multivitamin (*n* = 4), iron (*n* = 2), high energy (*n* = 1), protein powder (*n* = 1), B vitamins (*n* = 2), Vitamin C (*n* = 3), Vitamin E (*n* = 1), garlic (*n* = 2) and calcium (*n* = 2)

Note: AN denotes anorexia nervosa; BN denotes bulimia nervosa; EDNOS denotes eating disorders not otherwise specified; IQR denotes interquartile range; BMI denotes body mass index; BMR denotes basal metabolic rate; EEE denotes estimated energy expenditure; EEI denotes estimated energy intake; nutrient intakes include supplement usage

After energy-adjustment, dietary cholesterol via diet history and serum triglycerides showed moderate agreement (simple kappa K = 0.56, *p* = 0.04), and dietary iron and serum TIBC showed moderate-good agreement (simple kappa K = 0.48, *p* = 0.04; weighted kappa K = 0.68, *p* = 0.03) (Additional File 1 Table 5). For Spearman rank correlations, dietary iron and serum TIBC were significantly positively correlated following energy-adjustment only when supplements were included (*r* = 0.89, *p* = 0.02) (Table 2).

**Table 2:** Spearman’s rank correlation coefficients for diet history dietary intake and nutritional biomarkers with and without dietary supplements (*n* = 13)

Dietary intake	Nutritional biomarker	Correlation with Nutrients Excluding Dietary Supplements				Correlation with Nutrients Including Dietary Supplements			
		Crude		Energy Adjusted		Crude		Energy Adjusted	
		*r*	*p*	*r*	*p*	*r*	*p*	*r*	*p*
Saturated fat	Cholesterol	0.23	0.61	-0.14	0.76	0.23	0.61	-0.14	0.82
	Triglycerides	-0.40	0.60	0.80	0.20	-0.40	0.60	0.40	0.60
Cholesterol	Cholesterol	0.23	0.61	0.29	0.53	0.23	0.61	0.29	0.53
	Triglycerides	-0.40	0.60	-0.40	0.60	-0.40	0.60	-0.40	0.60
Protein	Protein	-0.41	0.16	0.38	0.20	-0.41	0.16	0.38	0.20
	Albumin	-0.63	0.02*	0.17	0.57	-0.63	0.02*	0.17	0.57
Iron	Iron	-0.11	0.82	0.71	0.07	-0.11	0.82	-0.11	0.82
	Haemoglobin	0.43	0.40	-0.26	0.62	0.43	0.40	-0.26	0.62
	Ferritin	-0.53	0.14	0.07	0.86	-0.53	0.14	0.13	0.73
	TIBC	0.94	< 0.01^*^	0.60	0.21	0.94	< 0.01*	0.89	0.02*
Folate	Red cell folate	0.66	0.16	0.49	0.33	0.66	0.16	0.77	0.07

Note: TIBC denotes total iron-binding capacity; Statistically significant findings (*p* < 0.05) are marked with *

For the Bland-Altman analyses, dietary protein and serum albumin showed that most points lie within the range of the mean ± 2SD and there was a small variation around the mean difference (LOA: -2.47 to 1.55) indicating some error (Additional File 1 Fig. 2). The trend suggests as protein intake increases, the size of the difference between the 2 measures decreases. A small variation existed around the mean difference (LOA: − 0.81 to 2.6) for dietary iron with serum TIBC, indicating some error in the diet history method of measuring iron. The trend suggests as intake increases, the size of the difference between the two measures decreases. Additional File 1 depicts the Bland-Altman analyses.

## Discussion

This pilot study offers a useful contribution to the limited knowledge of the validity of the diet history assessment method in the context of eating disorders, where there is currently a paucity of evidence. Findings suggest that the diet history may be a useful dietary assessment method for assessing dietary cholesterol and iron intakes in people with an eating disorder, and that accuracy in measurement of dietary protein and iron increases as dietary intake increases (the larger the intake the more accurate the measurement). Other findings were the importance of including supplement use in dietary assessments and consideration of comparison methods in eating disorders dietary validation studies. Comparison of these findings to other eating disorders dietary measurement studies is limited due to differences in diagnostic groups included, test or comparison method used, analyses conducted and nutrients selected for validation [9–13].

Administration and interpretation of diet histories in eating disorders should take into account the potential impact of disordered eating behaviour on measurement error, and the importance of targeted questioning specific to eating disorders. The use of standardized protocols and training in diet history administration in the context of eating disorders is warranted to minimize systematic biases. Binge eating episodes and cognitive changes associated with starvation may pose challenges for both administration and participant recall, although preoccupation with food intake may potentially aide recall. Targeted questioning and responding around particular nutrients which may be inadequate or consumed in excessive amounts, specific food items, serve sizes, dietary practices, food preparation practices, missed meals, periods of dietary restriction and binge eating episodes would be important. The use of food models may improve reporting. Around half of the participants consumed dietary supplements and usage influenced dietary assessment accuracy, highlighting the importance of targeted questioning around supplement use in diet history administration. Herbal, vitamin, mineral and other nutritional supplements can provide concentrated sources of nutrients and are important to assess accurately. Administration by trained dietitians can assist with the accuracy of dietary recall methods.

Strengths of this study include using routine clinical data collected as part of a standard nutritional assessment and use of biomarkers of nutritional status as an objective comparison method. There are many limitations to this study. The sample size is small and although this may limit the power to detect further relationships, results should be interpreted with caution. The small sample size also limited the ability to assess the impact of eating disorder characteristics on the accuracy of the diet history. The small number of nutritional biomarkers available limits broader conclusions regarding diet history validity in assessing a range of macronutrient and micronutrient intakes. Protein, albumin, cholesterol and folate can also be affected by many non-dietary factors, which weakens their utility as a comparison measure [14, 18]. Dietary iron was found to positively correlate with TIBC. Negative correlation was anticipated and results were influenced by dietary supplement usage, suggesting further in-depth studies are required to explore this finding in the context of supplement use and disordered eating behaviors.

Further research determining the validity and use of the diet history and other dietary assessment tools suitable for use in eating disorders is required. Larger sample sizes to account for individual variance is important. People with an eating disorder can have minimal day-to-day variability in dietary intake or have significant variability due to patterns of dietary restriction, binge eating episodes and supplement use, posing a challenge to dietary assessment. The use of pre-existing routine blood tests as a comparison method limited the extent of diet history validation that could be explored. There is emerging research on biomarkers of intake of specific foods or food groups (as opposed to nutrients), and future studies could broaden nutritional biomarker selection [19]. Consideration of suitable validation comparison methods that are cost-effective, reduce burden on participants and allow measurement of dietary intake in naturalistic settings as part of routine care are required.

This pilot study adds to the small body of literature addressing the accuracy of self-report dietary assessment methodologies in eating disorders. The diet history may be a useful clinical tool in assessing dietary intake in people with an eating disorder, particularly for dietary cholesterol, iron and at larger intakes protein. Further research is required to determine bias and error of a range of dietary assessment methods in different ages, diagnoses and settings. This could inform the use of dietary assessment methods in clinical practice and the development of training and standardization of procedures for dietary assessment methods.

## Supplementary Information

Below is the link to the electronic supplementary material.

Additional File 1: Additional methods and results (Additional File 1 contains additional methodology and results for readers wishing to read further)

## Data Availability

The datasets generated and/or analysed during the current study are not publicly available due to the small sample size and participant privacy, though are available from the corresponding author on reasonable request.

## Abbreviations

AN: Denotes anorexia nervosa
BMR: Denotes basal metabolic rate
BN: Denotes bulimia nervosa
EDNOS: Denotes eating disorders not otherwise specified
EEE: Denotes estimated energy expenditure
EEI: Denotes estimated energy intake
IQR: Denotes interquartile range
PAL: Denotes physical activity level
SD: Denotes standard deviation
TIBC: Denotes total iron-binding capacity
