# Supplementary material for: Dietary assessment methodologies in eating disorders: a pilot comparative validation study exploring the diet history method
Source: J Eat Disord. 2025 Sep 29;13:215. doi: 10.1186/s40337-025-01392-x (PMC12482415; doi:10.1186/s40337-025-01392-x)
Supplement: Supplementary file 1 — Additional File 1: Additional methods and results (Additional File 1 contains additional methodology and results for readers wishing to read further) [file 40337_2025_1392_MOESM1_ESM.pdf]

Additional File 1: Additional methodology and results

Dietary assessment methodologies in eating disorders: a pilot validation study exploring the diet history method

Body mass index (BMI) was calculated using weight (kg)/height $(m)^2$. Estimated average physical activity level (PAL) and Basal Metabolic Rate (BMR) were calculated according to Warwick (1990). BMR was multiplied by PAL to determine Estimated Energy Expenditure (EEE). Diet histories were administered by an experienced eating disorders dietitian and analysed using Foodworks Nutrition Analysis Program Version 3.02 by Xyris Software (2009), Brisbane, Australia, based on the Australian Nutrient database (NUTTAB 1995). Participants were asked about specific types, quantities and timing of eating, periods of dietary restriction or fasting, binge eating episodes and use of nutritional supplements. Patients of the service were requested to complete routine blood tests within one week before the initial assessment (conducted by local pathology companies).

Energy-adjusted nutrients from diet histories were calculated using the method described by Willet (1998; 1997), including calculating residuals from the regression model with total caloric intake (independent) and absolute nutrient intake (dependent variable). Spearman’s rank correlation coefficients examined the relationship between nutrient intakes and nutritional biomarkers, before and after energy intake adjustment. Simple and quadratic weighted kappa statistics were calculated for crude and energy-adjusted nutrients, to compare tertiles of intake for each nutrient from diet histories and biomarkers. The Bland-Altman method was used to assess agreement between the diet history and biomarkers by plotting the difference between the two measurements against the average of the two measurements (Flood, 2004). Limits of agreement (LOA, 95% confidence intervals) were set at 2 standard deviations (SD) from the mean bias. The diet history and biomarker measurements were considered in agreement if bias calculations fell between these limits.

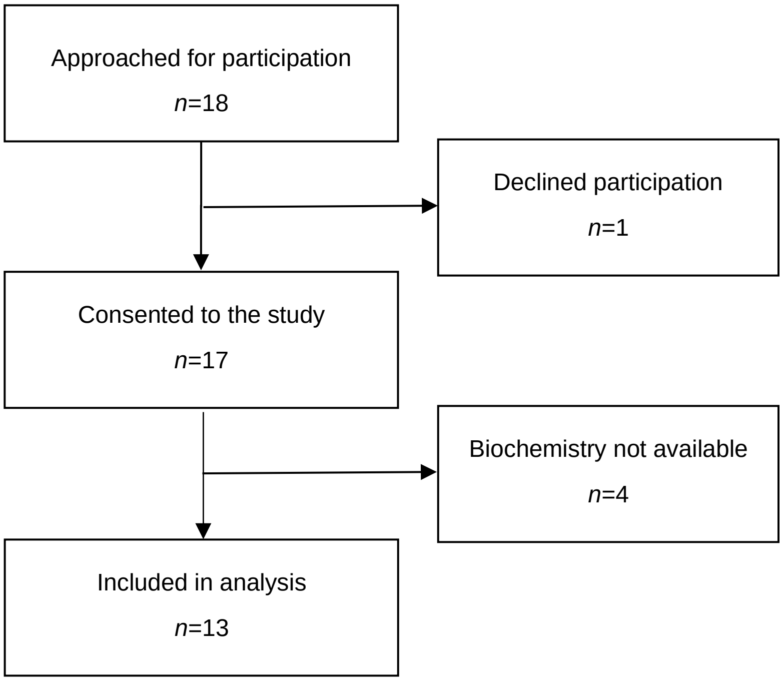

Figure 1: recruitment flowchart for the pilot study

Table 3 – Participant dietary supplement usage by diagnosis (*n*=13)

	AN and BN (*n*=5)	EDNOS (*n*=8)	All participants (*n*=13)
Supplement Type			
Multivitamin *n* (%)	1 (20)	2 (25)	3 (23)
Iron *n* (%)	0 (0)	2 (25)	2 (15)
High Energy (Ensure) *n* (%)	1 (20)	0 (0)	1 (8)
Protein powder *n* (%)	0 (0)	1 (13)	1 (8)
*Other *n* (%)	2 (40)	2 (25)	4 (31)

Note: AN denotes anorexia nervosa; BN denotes bulimia nervosa; EDNOS denotes eating disorders not otherwise specified. *Other: supplements not directly relating to the nutrients under this study (eg Vitamin E, garlic, calcium, B vitamins, Vitamin C).

Table 5 - Simple and quadratic weighted kappa statistics for crude and energy-adjusted nutrients by tertile (*n*=13)

Nutrient	Nutritional Biomarker	Crude Simple Kappa	*p*	Adjusted Simple Kappa	*p*	Crude Weighted Kappa	*p*	Adjusted Weighted Kappa	*p*
Saturated fat	Cholesterol	0.15	0.28	-0.09	0.64	0.07	0.37	0.00	0.50
	Triglycerides	0.27	0.20	0.20	0.29	0.00	0.50	0.50	0.16
Cholesterol	Cholesterol	0.56	0.02*	0.32	0.11	0.00	0.50	0.57	0.06
	Triglycerides	-0.45	0.92	0.56	0.04*	-0.40	0.80	0.67	0.05
Protein	Protein	0.07	0.36	0.19	0.17	-0.25	0.82	0.38	0.09
	Albumin	0.07	0.36	0.30	0.06	-0.63	0.99	0.25	0.18
Iron	Iron	0.34	0.09	-0.13	0.69	0.25	0.24	0.29	0.22
	Haemoglobin	0.25	0.19	0.25	0.18	0.25	0.27	0.25	0.25
	Ferritin	0.00	0.50	-0.17	0.77	-0.36	0.89	0.00	0.50
	TIBC	0.74	0.01*	0.48	0.04*	0.84	0.02*	0.68	0.03*
Folate	Red cell folate	0.25	0.14	0.00	0.50	0.40	0.14	0.50	0.11

Note: TIBC denotes total iron-binding capacity. Statistically significant findings ($p<0.05$) are marked*.

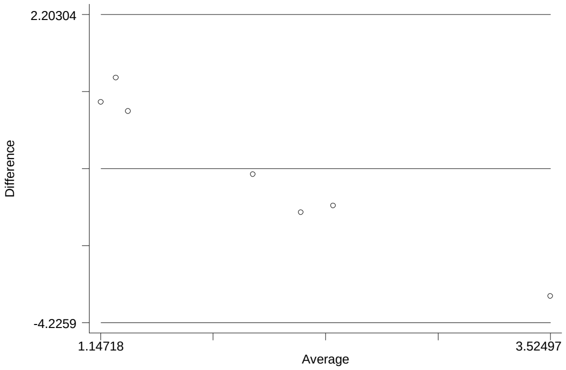

a. Saturated Fat & Serum Cholesterol

Bland-Altman comparison of tSChol and tDHxSupSatFat
Limits of agreement (Reference Range for difference): -4.226 to 2.203
Mean difference: -1.011 (CI -2.498 to 0.475)
Range : 1.147 to 3.525
Pitman's Test of difference in variance: r = -0.981, n = 7, p = 0.178

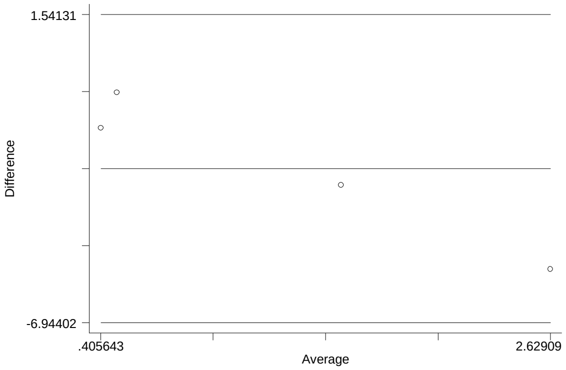

b. Saturated Fat & Serum Triglycerides

Bland-Altman comparison of tSTG and tDHxSupSatFat
Limits of agreement (Reference Range for difference): -6.944 to 1.541
Mean difference: -2.701 (CI -6.077 to 0.674)
Range : 0.406 to 2.629
Pitman's Test of difference in variance: r = -0.974, n = 4, p = 0.428

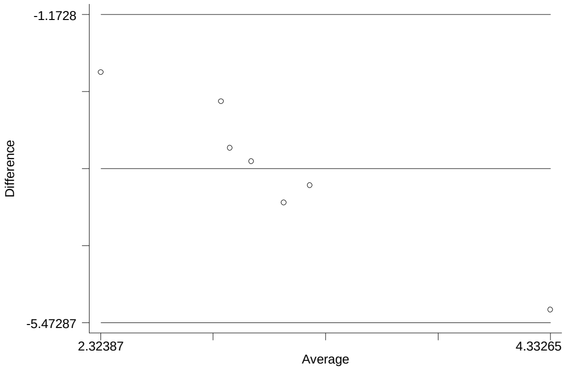

c. Cholesterol & Serum Cholesterol

Bland-Altman comparison of tSChol and tDHxSupChol
Limits of agreement (Reference Range for difference): -5.473 to -1.173
Mean difference: -3.323 (CI -4.317 to -2.329)
Range : 2.324 to 4.333
Pitman's Test of difference in variance: r = -0.966, n = 7, p = 0.181

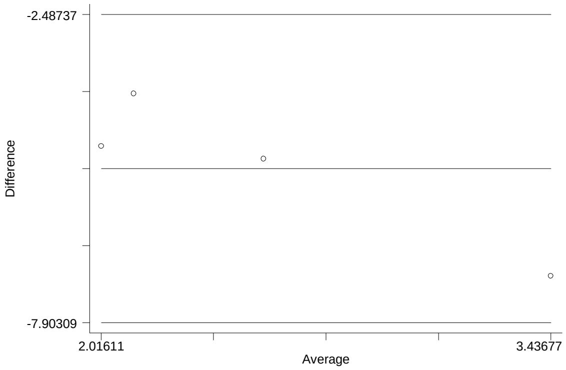

d. Cholesterol & Serum Triglycerides

Bland-Altman comparison of tSTG and tDHxSupChol
Limits of agreement (Reference Range for difference): -7.903 to -2.487
Mean difference: -5.195 (CI -7.350 to -3.041)
Range : 2.016 to 3.437
Pitman's Test of difference in variance: r = -0.935, n = 4, p = 0.436

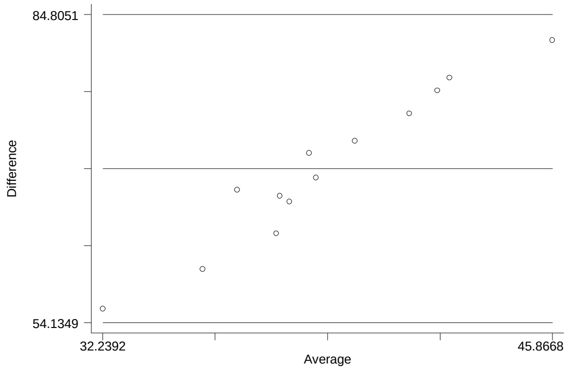

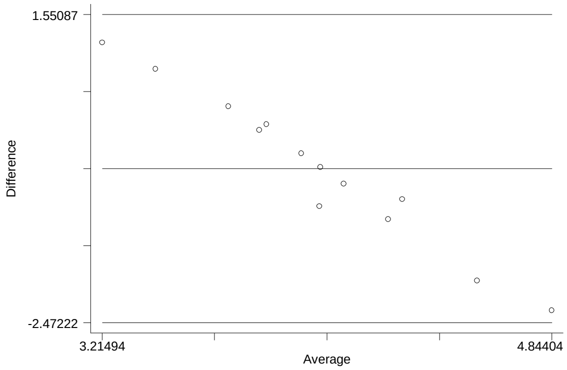

e. Protein & Serum Protein

Bland-Altman comparison of sprot and tDHxSupProt
Limits of agreement (Reference Range for difference): 54.135 to 84.805
Mean difference: 69.470 (CI 64.837 to 74.103)
Range : 32.239 to 45.867
Pitman's Test of difference in variance: r = 0.970, n = 13, p = 0.000

f. Protein & Serum Albumin

Bland-Altman comparison of tSAlb and tDHxSupProt
Limits of agreement (Reference Range for difference): -2.472 to 1.551
Mean difference: -0.461 (CI -1.068 to 0.147)
Range : 3.215 to 4.844
Pitman's Test of difference in variance: r = -0.983, n = 13, p = 0.040

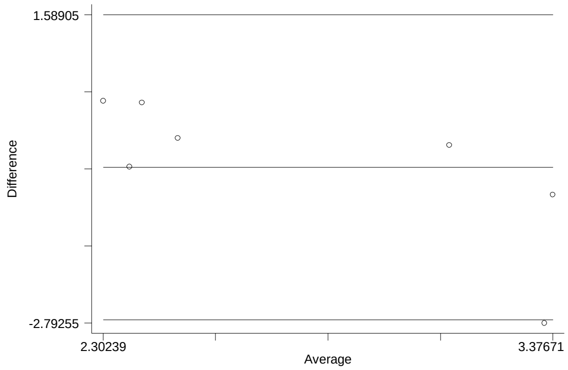

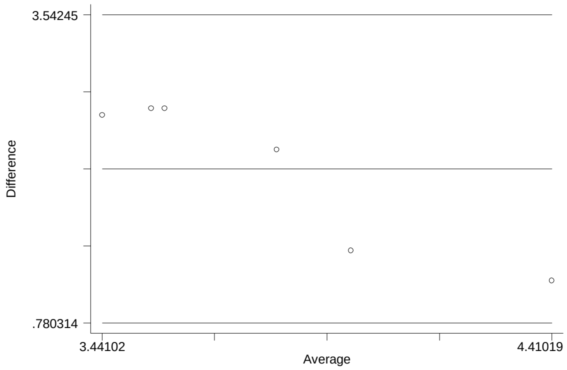

g. Iron & Serum Iron

Bland-Altman comparison of tSFe and tDHxSupFe
Limits of agreement (Reference Range for difference): -2.750 to 1.589
Mean difference: -0.580 (CI -1.584 to 0.423)
Range : 2.302 to 3.377
Pitman's Test of difference in variance: r = -0.730, n = 7, p = 0.245

h. Iron with Serum Haemoglobin

Bland-Altman comparison of tSHb and tDHxSupFe
Limits of agreement (Reference Range for difference): 0.780 to 3.542
Mean difference: 2.161 (CI 1.437 to 2.886)
Range : 3.441 to 4.410
Pitman's Test of difference in variance: r = -0.941, n = 6, p = 0.242

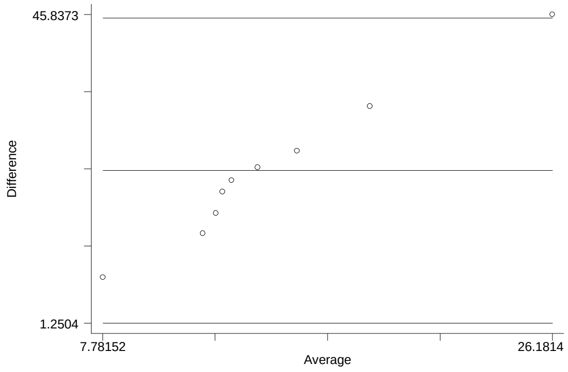

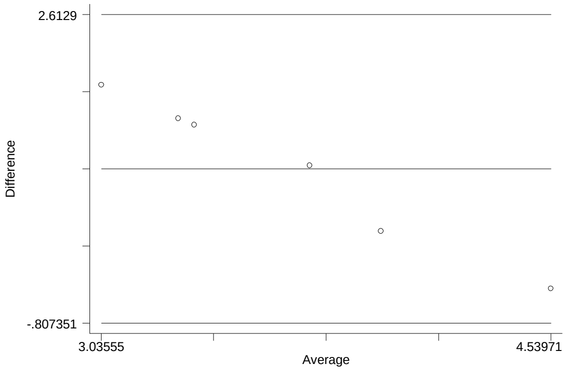

i. Iron with Serum Ferritin

Bland-Altman comparison of sferr and tDHxSupFe
Limits of agreement (Reference Range for difference): 1.250 to 45.355
Mean difference: 23.303 (CI 14.827 to 31.778)
Range : 7.782 to 26.181
Pitman's Test of difference in variance: r = 0.987, n = 9, p = 0.000

j. Iron with Serum TIBC

Bland-Altman comparison of tSTIBC and tDHxSupFe
Limits of agreement (Reference Range for difference): -0.807 to 2.613
Mean difference: 0.903 (CI 0.005 to 1.800)
Range : 3.036 to 4.540
Pitman's Test of difference in variance: r = -0.991, n = 6, p = 0.232

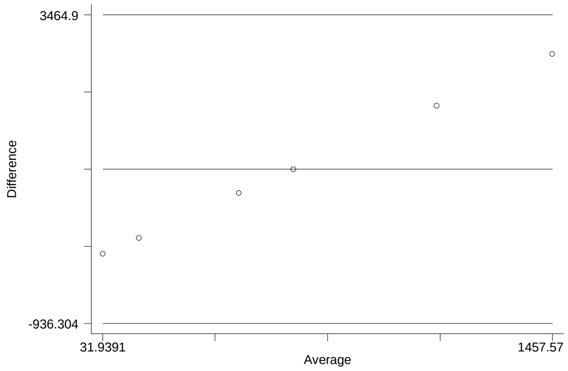

k. Folate with Red Cell Folate

Bland-Altman comparison of sfol and tDHxSupFol
Limits of agreement (Reference Range for difference): -936.304 to 3464.902
Mean difference: 1264.299 (CI 109.603 to 2418.995)
Range : 31.939 to 1457.571
Pitman's Test of difference in variance: r = 1.000, n = 6, p = 0.000

Figure 2: Bland-Altman analyses for dietary intake and nutritional biomarkers

References:

Flood, V. M., Smith, W. T., Webb, K. L., & Mitchell, P. (2004). Issues in assessing the validity of nutrient data obtained from a food-frequency questionnaire: folate and vitamin B12 examples. *Public Health Nutr*, *7*(6), 751-756.

Warwick, P. M. (1990). Predicting Food Energy Requirements from Estimates of Energy Expenditure. In *Recommended Nutrient Intakes: Australian Papers*. Australian Professional Publications.

Willet, W., & Lenart, E. (1998). Reproducibility and Validity of Food Frequency Questionnaires. In *Nutritional Epidemiology*. Oxford University Press.

Willett, W. C., Howe, G. R., & Kushi, L. H. (1997). Adjustment for total energy intake in epidemiologic studies. *Am J Clin Nutr*, *65*(4 Suppl), 1220S-1228S; discussion 1229S-1231S. https://doi.org/10.1093/ajcn/65.4.1220S

Xyris Software (Australia) Pty Ltd. (2009). *Foodworks Professional*. In
